# Three-Layered Annular Plate Made of Functionally Graded Material Under a Static Temperature Field

**DOI:** 10.3390/ma17225484

**Published:** 2024-11-10

**Authors:** Dorota Pawlus

**Affiliations:** Faculty of Mechanical Engineering and Computer Science, University of Bielsko-Biala, 43-308 Bielsko-Biala, Poland; doro@ubb.edu.pl

**Keywords:** composite FGM, three-layered annular plate, temperature field, critical state, finite-difference method

## Abstract

The presented problem considers the static temperature analysis of a three-layered, annular plate with heterogeneous facings made of material with radially variable parameters. They are defined by the accepted exponent functions. The plate is composed of thin metal facings and a thicker foam core. The plate is loaded with a flat temperature field with a gradient directed across the plate radius. Using the approximation finite-difference method, the eigen-value problem is solved in order to calculate the temperature differences between plate edges, which cause a loss of plate stability. Taking into account the different material and geometrical parameters, the critical temperature state parameters are evaluated. The meaning of the mixed system of parameters connected with the plate shape geometry, dimensions of the plate-transversal structure, and with the gradation of the material in the radial direction on the thermal response of the composite plate have been found. Numerous results of numerical calculations show the responses of the examined composite plate with facings made of the heterogeneously directed material.

## 1. Introduction

Heterogeneous materials for layers of composite plates enable the creation of new structures designed for selected applications. Different material parameters, in combination with the suitable mass arrangement, create distinct elements whose unique mechanical and thermal loading properties can be predicted. For such systems, the action of the temperature field can be controlled. An example is the response of the annular plate subjected to the temperature gradient between the edges. Various applications of annular plates, for example, in mechanical, civil, or nuclear engineering and also the aerospace industry, require a search for new solutions that are appropriate for a given application. 

We propose a three-layered annular plate with functionally graded material (FGM) for the facings in this paper. Thermal loading influences plate stability since it changes the geometrical parameters and load capacity of the plates. The main aim of the presented investigations is to recognize the plate stability response to the static temperature field. The building of a new plate structure with selected FGM layers—i.e., the facings—is the novel element that widens our understanding of composite, heterogeneous plates in various conditions. Applications could include cases where the annular plate experiences thermal loads from the temperature fields around the plate’s inner and outer edges. 

A selected analysis, which considers the vibration, buckling, and thermal loading of multi-material annular plates, is presented. This work exhibits an extensive range of studies and the application of different calculation techniques. A vibration and buckling analysis of a sandwich square plate with an FGM metal plate core has been presented in a previous work [1]. Here, the influence of geometry, material gradation, and the system of boundary conditions have been taken into account. The gradation law changes the transversal geometry of the distribution of the core metal material. The plate-buckling response was shown, which is dependent on an acceptable analysis of the plate parameters. 

The influence of graphene platelets, which transversally reinforce the composite annular plate that is located on the elastic foundation, on its stability is presented in a previous work [2]. The influence of the geometry parameters of graphene dispersions on plate thermal responses has been examined in detail. A functionally graded graphene platelet reinforced with a nanocomposite annular plate subjected to thermal loading was examined in earlier research [3]. Here, the asymmetric stability problem was analyzed. The transversal plate structure consists of functionally graded graphene laminas that are differently orientated. A detailed discussion was conducted on parameters for analyzing the plates in various thermal environments. 

The axisymmetric thermal buckling of FGM annular plates was analyzed in an earlier paper [4]. A plate with variable thickness was thermally loaded across the plate-radial coordinate. The effects of the geometrical, boundary, and thermal loading were studied. The problem was solved using the finite elements. The influence of the Winkler foundations, geometry, and material parameters on functionally graded piezoelectric annular plates has also been investigated [5]. The problem of the three-dimensional free vibrations was solved semi-analytically. FGM annular plates under rapid heating were also examined [6]. The influence of thermal shock on plate vibrations depending on various parameters connected with loading, plate geometry, and boundary conditions was analyzed. This work confirmed the significant effect of these parameters on the thermal response, especially for the thin plates.

In addition, the frequency response of the annular sector and sector plates for various FG materials has been subject to examination [7]. The effect of different geometric, material, and boundary parameters on frequency values was determined. An analytical study on the buckling of FGM annular plates with an elastic foundation is presented in ref. [8]. Using classical plate theory, the three models for thermal loading were analyzed for the calculational process of the critical temperature of plates with various parameters. The FGM annular plates under thermo-mechanical loads in the large deflection problem were also examined in the literature [9]. The von Karman plate theory was used to analyze the effect of plate parameters. 

There is a special group of problems that consider heterogeneous plates, shells with periodic geometry, or/and material distribution. Different problems, such as stability, thermoelasticity, heat conduction, and temperature dependence, are solved using tolerance-modeling techniques. Selected works have undertaken such investigations [10,11,12,13,14,15,16]. The proposed methods for the solving of complex, multiparameter tasks reveal the special behavior and detailed parameters of the microstructure elements, which constitute the examined composite materials. Requiring the most attention is the key feature for the functional gradation of materials, which is porosity. Models of sandwich elements porously, which are heterogeneously subjected to thermal loading, are presented in previous works [17,18]. Here, the effect of the porosity of the functionally graded materials on thermal resistance and strength capacity was shown. 

In this paper, we present the problem of an annular plate that is composed of three layers, among which are two outer ones that are made of FGMs; this configuration has now entered into a wider group of actual problems that have been undertaken in numerous works. The action of the specially targeted temperature field on the FGM plate facings causes a buckling deformation of the plate structure for corresponding values of the critical temperature differences between the plate edges. The presented investigation shows the responses of the heterogeneous, annular plates due to the temperature fields occurring around them. The temperature-critical difference, Δ*T_cr_*, between the plate edges is a main parameter in the presented evaluation. It identifies the static, critical state of the thermally loaded plate. The static problems are solved semi-analytically with the use of the finite-difference method. Numerous results show the temperature gradient effect on the annular plate response and the participation of the nonhomogeneous facings in its composite structure. 

The presented approach is a new proposal to evaluate the annular response of composite plates whose outer facings are not homogeneous. The special composition of the plate-transversal structure, where only the metal facings are made of materials with functional gradation, creates new possibilities for applications. The presented results show the sensitivity of complex plate structures with temperature differences, which can exist between plate perimeters, acting upon them. Detailed values of the critical temperature differences, Δ*T_cr_*, particularly those that are minimal, indicate numbers that can cause plate buckling, a change in the plate preliminary geometry, and strength capacity. 

## 2. Problem Formulation

The three-layered, annular plate is the object of this analysis. The plate is subjected to temperature fields existing around the plate perimeter. The temperature difference between plate edges occurs. It causes the loss of plate stability; the problem is static. A three-layered plate is composed of thin metal facings and a thicker foam core. The facings are made of heterogeneous material composed of two components. The participation and radial distribution of each metal component are expressed by the value of *VV* parameter, which is calculated according to Equation (6) for the functionally graded material (FGM) facings.

**Figure 1:**
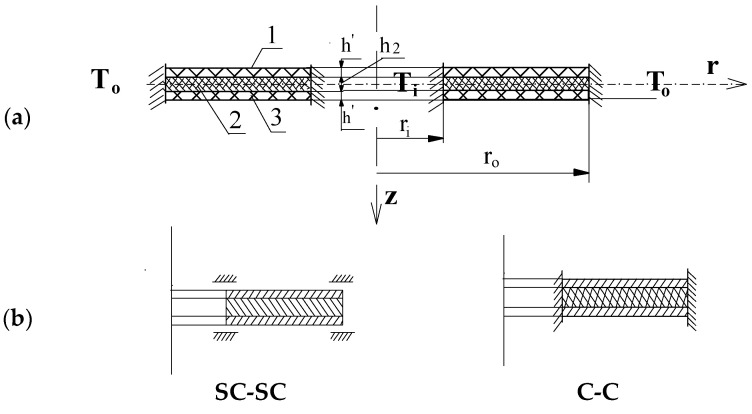
Scheme for the three-layered annular plate composed of facings (layers 1 and 3) and a core (layer 2): (**a**) subjected to the stationary, axisymmetrical temperature fields (*T_i_* and *T_o_*) and (**b**) with different support systems, i.e., SC-SC or C-C.

Both plate edges are clamped (C-C) or slidably clamped (SC-SC). The main analysis is focused on the case of the clamped–clamped annular plate. A scheme for the plate is presented in Figure 1. The plate core is made of polyurethane foam.

The Figures given in this paper present the distribution of the values of critical temperature differences, Δ*T_cr_*, versus the value of the plate mode m, which describes the form of the loss of the plate stability expressed by the number of buckling circumferential waves. The results are mainly obtained with the application of the approximation finite-difference method (FDM) during the analytical and numerical calculations. Some of the results are compared with the ones calculated for a model built with the usage of the finite-element method (FEM) [19].

The temperature field model is fixed, flat, and axisymmetric. There is no heat exchange between the plate layers in the transversal directions. The material parameters do not depend on the temperature values. The temperature difference between the plate edges expresses two kinds of thermal gradient: positive, where the inner temperature *T_i_* in the plate hole is higher than temperature *T_o_* around the outer plate perimeter (*T_i_* > *T_o_*), and a negative one, in which an opposite temperature distribution exists (*T_i_* < *T_o_*).

A temperature distribution in the plate radial direction was adopted for smoothly changing material parameters. In the radial plate direction, this distribution does not significantly differ from the one that determines the temperature-radial change for an isotropic plate. Next, the temperature *T* changes in the radial plate direction according to the logarithmic formulae presented in the following equation, which is based on the one presented in previous work [20], which centers on a long cylinder with a circular hole, and other papers [21,22]:

(1) $$T=T_{o}+\frac{T_{i}-T_{o}}{\ln\rho_{i}}\ln\rho$$

where $\rho =\frac{r}{r_{o}},\;\rho_{i}=\frac{r_{i}}{r_{o}}$ are dimensionless radius and inner plate radius, respectively.

## 3. Problem Solution

The solution is based on the equations for the annular three-layered plate, subjected to the stationary temperate field, which is presented, for example, in refs [23,24,25]. The technique for the solution of the annular plate stability problem is shown in detail in a previous work [26]. The main elements of the solution are given as follows:

- The system of the static equilibrium equations is established.
- The cross-section of the plate structure is described using the broken-line hypothesis.
- The linear physical relations of Hooke’s law are used to assess the stress state in the facings.

The relevant equations are expressed as follows:

(2) $$\sigma_{r_{1(3)}}=\frac{E_{r}}{1-\nu_{r}^{2}}(\varepsilon_{r_{1(3)}}+\nu_{r}\varepsilon_{\theta_{1(3)}})-\frac{E_{r}\alpha_{r}}{1-\nu_{r}}T\left(r,\theta ,z\right),$$

(3) $$\sigma_{\theta_{1(3)}}=\frac{E_{r}}{1-\nu_{r}^{2}}(\varepsilon_{\theta_{1(3)}}+\nu_{r}\varepsilon_{r_{1(3)}})-\frac{E_{r}\alpha_{r}}{1-\nu_{r}}T\left(r,\theta ,z\right),$$

where *E_r_* and *ν_r_* are Young’s modulus and Poisson’s ratio, respectively, which depend on the plate radius of the facing material; *α_r_* is the thermal expansion coefficient depending on the facing radius; and *T* is the temperature growth that changes in the plate radial direction (1). 

Here, the linear physical relations for the plate core layer are used, and the resultant membrane forces corresponding to radial, *N_r_*, and circumferential, *N_θ_*, normal forces and shear ones *T_rθ_* have been determined by the introduction of the stress function *Φ*. The transverse radial, *Q_r_*_2_, and circumferential forces, *Q_θ_*_2_, for the plate core, are expressed, and then the resultant forces, *Q_r_* and *Q_θ_*, of the whole plate are established.

After the algebraic operations, the following main equation that describes the plate deflections can be presented, i.e.,

(4) $$\begin{array}{l} k_{1}w_{\prime rrrr}+\frac{2k_{1}}{r}w_{\prime rrr}-\frac{k_{1}}{r^{2}}w_{\prime rr}+\frac{k_{1}}{r^{3}}w_{\prime r}+\frac{k_{1}}{r^{4}}w_{\prime \theta \theta \theta \theta }+\frac{2(k_{1}+k_{2})}{r^{4}}w_{\prime \theta \theta }+\frac{2k_{2}}{r^{2}}w_{\prime rr\theta \theta }\\ -\frac{2k_{2}}{r^{3}}w_{\prime r\theta \theta }-G_{2}\frac{H^{\prime }}{h_{2}}\frac{1}{r}\left(\gamma_{\prime \theta }+\delta +r\delta_{\prime r}+H^{\prime }\frac{1}{r}w_{\prime \theta \theta }+H^{\prime }w_{\prime r}+rH^{\prime }w_{\prime rr}\right)=\\ \frac{2h^{\prime }}{r}\left(\frac{2}{r^{2}}\Phi_{\prime \theta }w_{\prime r\theta }\right.\;-\;\frac{2}{r}\Phi_{\prime \theta r}w_{\prime \theta r}+\frac{2}{r^{2}}w_{\prime \theta }\Phi_{\prime \theta r}-\frac{2}{r^{3}}\Phi_{\prime \theta }w_{\prime \theta }+w_{\prime r}\Phi_{\prime rr}+\Phi_{\prime r}w_{\prime rr}\\ \left.+\frac{1}{r}\Phi_{\prime \theta \theta }w_{\prime rr}+\frac{1}{r}\Phi_{\prime rr}w_{\prime \theta \theta }\right)\; \end{array}$$

where *k*_1_ = 2*D*, *k*_2_ = 4*D_rθ_* + *νk*_1_, *δ* = *u*_3_ – *u*_1_, *γ* = *v*_3_ – *v*_1_, H^’^ = *h*^’^ + *h*_2_, $D=\frac{E_{r}h^{3}}{12\left(1-\nu_{r}^{2}\right)}$ is the plate rigidity, $D_{r\theta }=\frac{G_{r}h^{3}}{12}$ is the flexural rigidity of the facings, *G*_2_ is the Kirchhoff’s modulus of plate core, *h*′ and h_2_ are the thickness of the plate outer layer and core, respectively, *u*_1(3)_ is the displacements of the points of the middle plane of facings in the radial direction, *v*_1(3)_ is the displacements of the points of the middle plane of facings in the circumferential direction, and w is the plate deflection.

The finite-difference method (FDM) has been used in the approximation process of the derivatives with respect to *ρ* via the central differences in the discrete points. Solving the eigenvalue problem involves the critical temperature difference Δ*T_cr_* calculated as the minimal value of Δ*T* from the following equation:

(5) $$\det\left(M_{APDG}-\Delta T\;M_{ACT}\right)=0,$$

where **M***_APDG_* and **M***_ACT_* are the matrices of the elements composed of geometric and material parameters of the plate, respectively, the quantity *b* is the length of the interval in the finite-difference method, the number *m* is the number of buckling waves, the stress function *Φ* has been expressed for the C-C and SC-SC plate models treating it as a solution to the disk state, and $\Delta T=T_{i}-T_{o}$ is a temperature difference.

## 4. FGM Model

Facing-material changes in the plate radial direction according to the power function can be represented by the following expression:

(6) $$VV={\left(\frac{r_{i}}{r_{i}-r_{o}}+\frac{r}{r_{o}-r_{i}}\right)}^{n},$$

where *r_i_* and *r_o_* are the inner and outer plate radius, *r* is the plate radius, and *n* is the number.

The character of the changes is modeled using Equation (6). The values of the plate’s inner and outer radii and the number of exponents *n* define the distribution of facing material. Material parameters, such as Young’s modulus E_r_, Kirchhoff’s modulus G_r_, Poisson ratio ν_r_, and linear expansion coefficient α_r_ of the plate material facings, are defined with the usage of Equation (6) according to the notation:

(7) $$W=W1+VV\left(W2-W1\right),$$

where *W*1 and *W*2 are the values of selected material parameters *E_r_*, G_r_, *ν_r_*, and *α_r_*, and *W* is the value of the material parameter of the facings, which is expressed for plate radius *r*.

**Figure 2:**
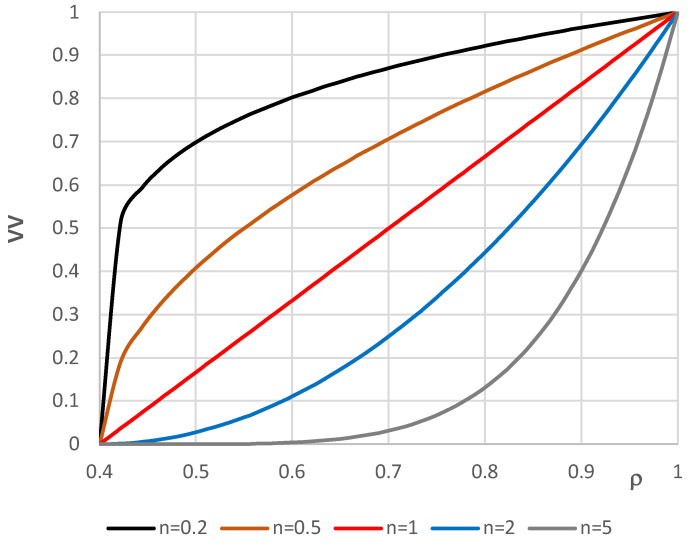
Distribution of the *VV* parameter versus plate radius *ρ.*

**Figure 3:**
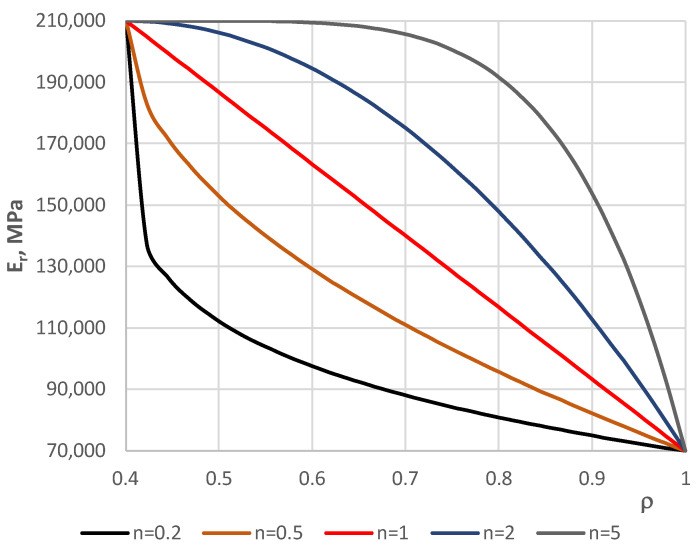
Distribution of Young’s modulus *E_r_* parameter versus plate radius *ρ.*

Figure 2 shows the distribution of the *VV* parameter for different plate radii. Figure 3 represents the values of Young’s modulus *E_r_*, expressed by *W* parameter (7), for different plate radii for five accepted numbers of exponent *n* equal to 0.2, 0.5, 1, 2, and 5. The facing material is built of two metals: steel and aluminum. The values of Young’s modulus for these two metals are equal to *E_ST_* = 210,000 MPa and *E_AL_* = 70,000 MPa, respectively. The curves show the participation of the two materials. The higher participation of steel is observed for *n* = 5 but much smaller for *n* = 0.2.

## 5. Plate Model Data

Exemplary calculations have been carried out for the selected geometrical and material plate parameters. All data are presented in Table 1. Both materials of the FGM plate facings and plate foam core are treated as elastic and isotropic. The plate is subjected to a positive or negative temperature gradient. 

## 6. Accuracy Assessment

**Figure 4:**
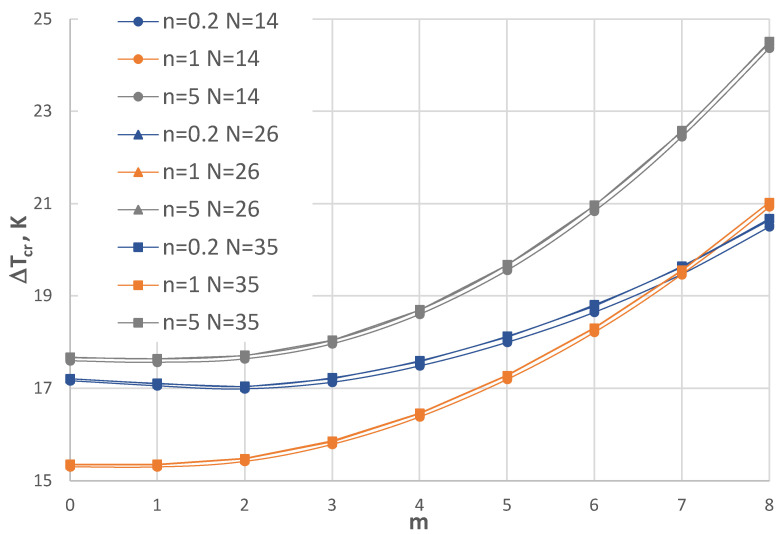
Distribution of the critical temperature difference Δ*T_cr_* versus mode m for the C-C plate models with *h*’ = 1 mm, *ρ_I_* = 0.4, and exponent number *n* = 0.2, 1, and 5 subjected to positive temperature gradient for three FDM discretization numbers, i.e., *N* = 14, 26, and 35.

**Figure 5:**
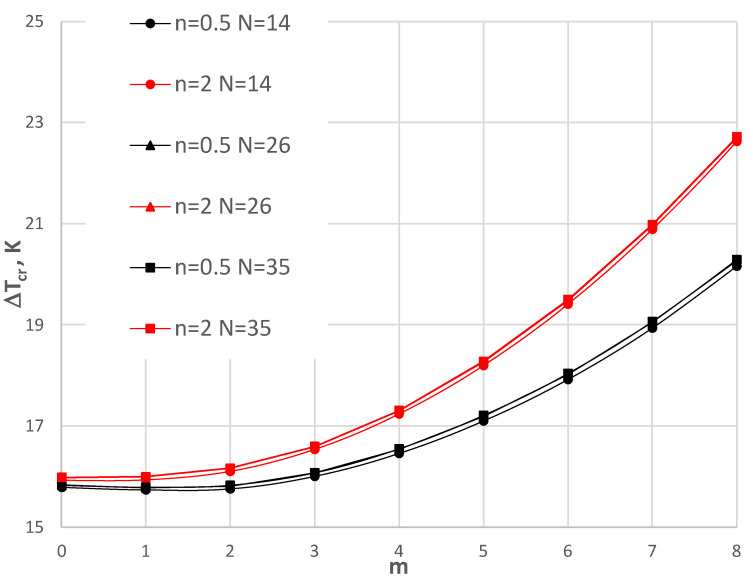
Distribution of critical temperature difference Δ*T_cr_* versus mode *m* for the C-C plate models with *h*’ = 1 mm, *ρ_i_* = 0.4 and exponent number *n* = 0.5 and 2 subjected to positive temperature gradient for three FDM discretization numbers, i.e., *N* = 14, 26, and 35.

**Figure 6:**
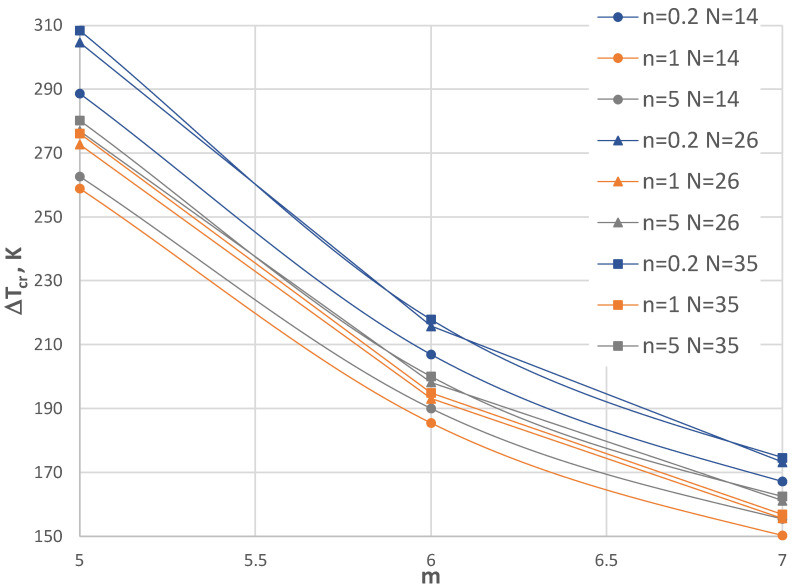
Distribution of critical temperature difference Δ*T_cr_* versus mode *m* for the SC-SC plate models with *h*’ = 1 mm, *ρ_i_* = 0.4, and exponent number *n* = 0.2, 1, and 5 subjected to negative temperature gradient for three FDM discretization numbers, i.e., *N* = 14, 26, and 35.

**Figure 7:**
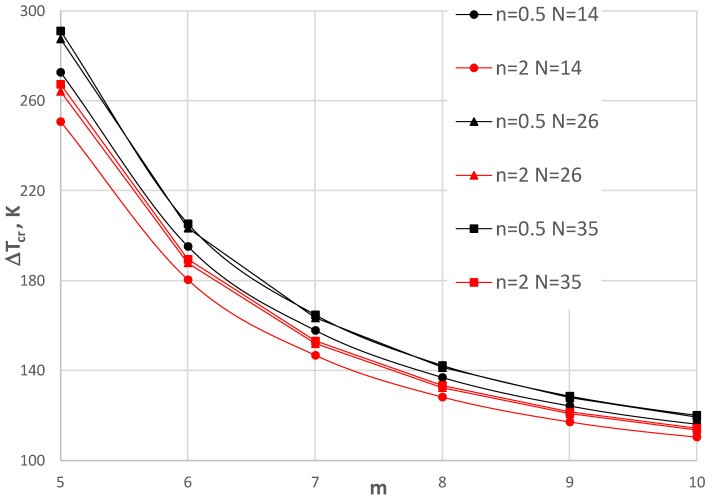
Distribution of critical temperature difference Δ*T_cr_* versus mode *m* for the SC-SC plate models with *h*’ = 1 mm, *ρ_i_* = 0.4, and exponent number *n* = 0.5 and 2 subjected to negative temperature gradient for three FDM discretization numbers, i.e., *N* = 14, 26, and 35.

The finite-difference method (FDM), which was used in the calculation process, requires an acceptance of the number of discretization points *N*. Figure 4, Figure 5, Figure 6 and Figure 7 show the comparison of values Δ*T_cr_* for the selected plate examples between three *N* numbers equal to 14, 26, and 35. Figure 4 and Figure 5 show the results for the C-C plate model with *h*’ = 1 mm and *ρ_i_* = 0.4 of the plate subjected to a positive temperature gradient. Figure 4 presents the comparison between the plates with the FGM facings defined by *n* = 0.2, 1, and 5. Figure 5 shows the comparison for plates with *n* = 0.5 and 2. Figs. 6 and 7 show the results for the SC-SC plate model with *h*’ = 1 mm and *ρ_i_* = 0.4 for a plate subjected to a negative temperature gradient. Similarly, Figure 6 presents the comparison between plates with FGM facings defined by *n* = 0.2, 1, and 5. Figure 7 shows the comparison for plates with *n* = 0.5 and 2. Table 2 presents selected values. It can be observed that there are small differences between the values calculated for N = 14, 26, and 35 for the C-C plate models. Greater differences exist between values Δ*T_cr_* obtained for the SC-SC plates calculated with numbers *N* = 14, 26, and 35. To increase the visibility of the presented diagrams, only selected *m* numbers have been chosen for the SC-SC plate models: *m* = 5–7 for the results presented in Figure 6 and *m* = 5–10 for the results shown in Figure 7. In general, the results show high accuracy in the precise range up to 5% of the technical error, which is accepted in numerical calculations of the values Δ*T_cr_* for *N* presented numbers of discrete points. A high accuracy is particularly observed for the C-C plate model. The number *N* = 26, which fulfills the convergence of values Δ*T_cr_* with number *N* = 35 for both the C-C and SC-SC plate models has been accepted in the FDM calculations. 

Table 3 presents the values of Δ*T_cr_* calculated using two kinds of plate models built using the FDM and FEM methods. Calculations for the homogeneous or heterogeneous three-layered plates are presented in an article by Pawlus [19]. The full annulus FEM plate model was built of shells and solid elements, which created a mesh of facings and the core. The calculations were carried out at the ACC CYFRONET in Cracow using the ABAQUS system (KBN/SGI_ORIGIN_2000/PŁódzka/030/1999). Presented in Table 3 is a comparison between the values of Δ*T_cr_*, which shows a very good agreement both for the C-C and SC-SC plate models. The plates were subjected to a positive temperature gradient. The facings are homogeneous, either made of steel or aluminum. Their thickness corresponds to *h*’ = 1 mm. The plate geometry is expressed by the inner radius *ρ_i_*, which is equal to 0.4. A good agreement is observed for both the axisymmetric form of the buckling (*m* = 0) and the asymmetric form with one circumferential wave (*m* = 1), as well as for the asymmetric form with several circumferential waves (*m* = 9 and 11). 

The underlined numbers represent the examples where the minimal value of ΔT_cr_ exists. Some differences for number *m* relating to the buckling waves are observed for both the C-C and SC-SC plate models. The numbers presented in brackets for the SC-SC FDM plate model show the values of Δ*T*_cr_ for plate mode number *m*, which is consistent with the mode of the FEM plate model.

## 7. C-C Plate Model

**Figure 8:**
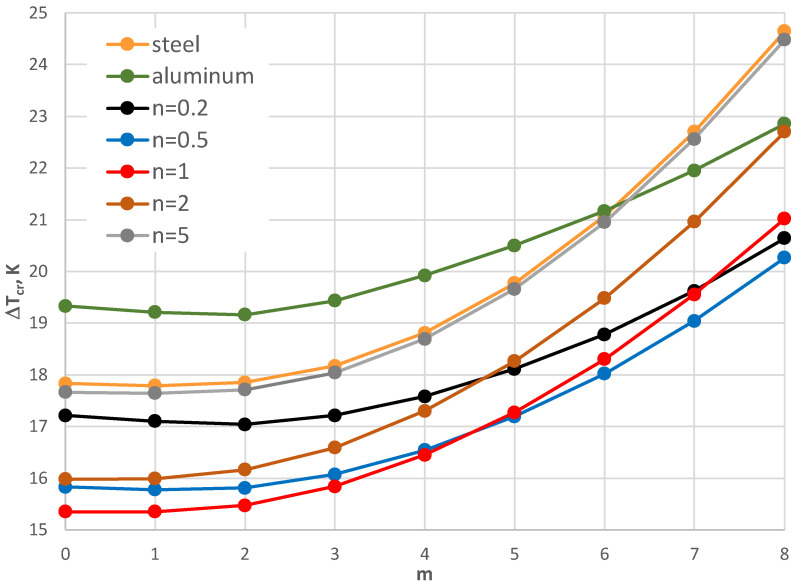
Distribution of the critical temperature difference Δ*T_cr_* versus mode *m* for *ρ_i_* = 0.4.

**Figure 9:**
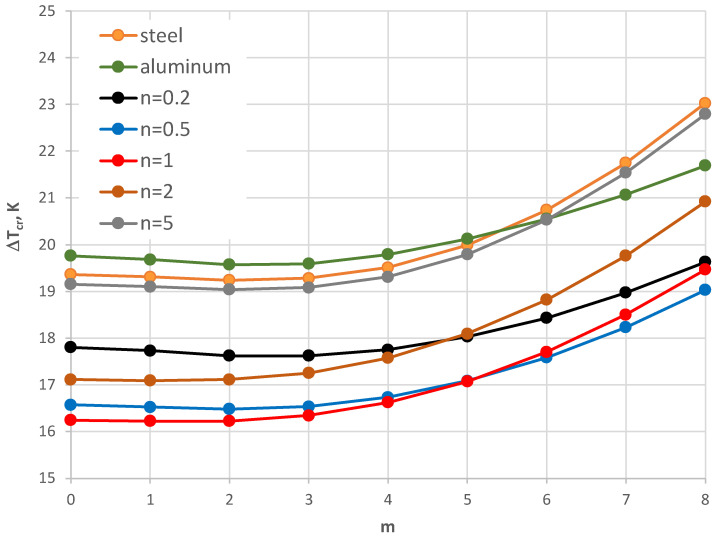
Distribution of the critical temperature difference Δ*T_cr_* versus mode *m* for *ρ_i_* = 0.5.

**Figure 10:**
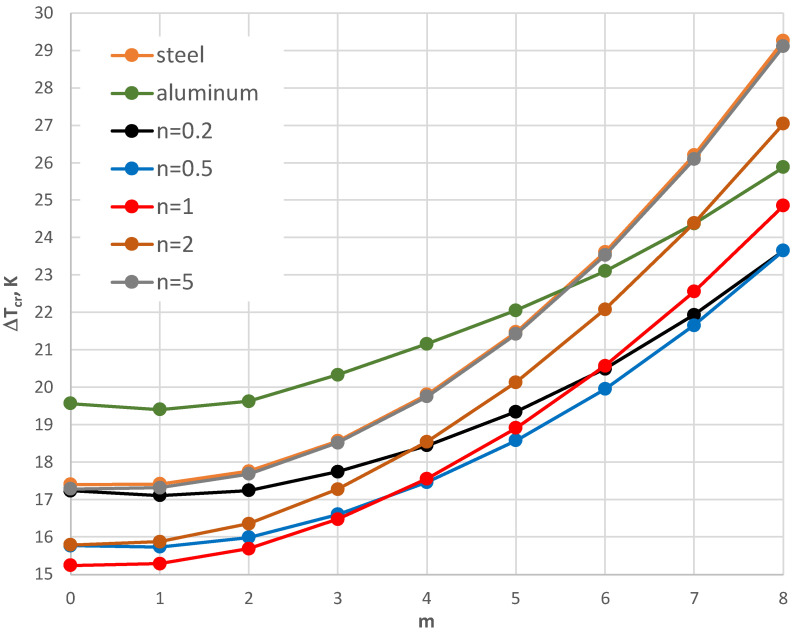
Distribution of the critical temperature difference Δ*T_cr_* versus mode *m* for *ρ_i_* = 0.3.

**Figure 11:**
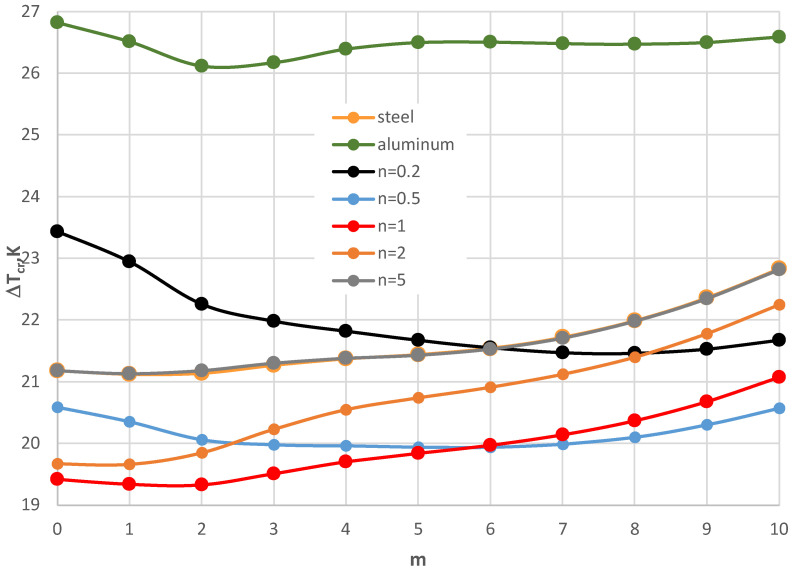
Distribution of the critical temperature difference Δ*T_cr_* versus mode *m* for *ρ_i_* = 0.4 and a facing thickness *h*’ = 0.5 mm.

**Figure 12:**
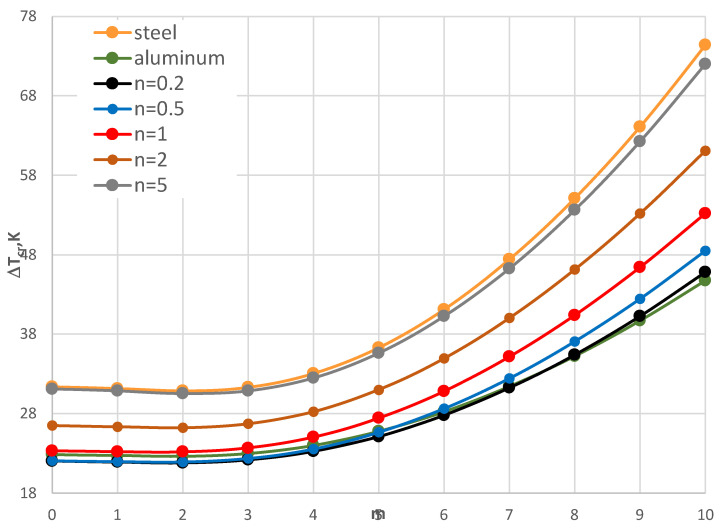
Distribution of the critical temperature difference Δ*T_cr_* versus mode *m* for *ρ_i_* = 0.4 and a facing thickness *h*’ = 2 mm.

**Figure 13:**
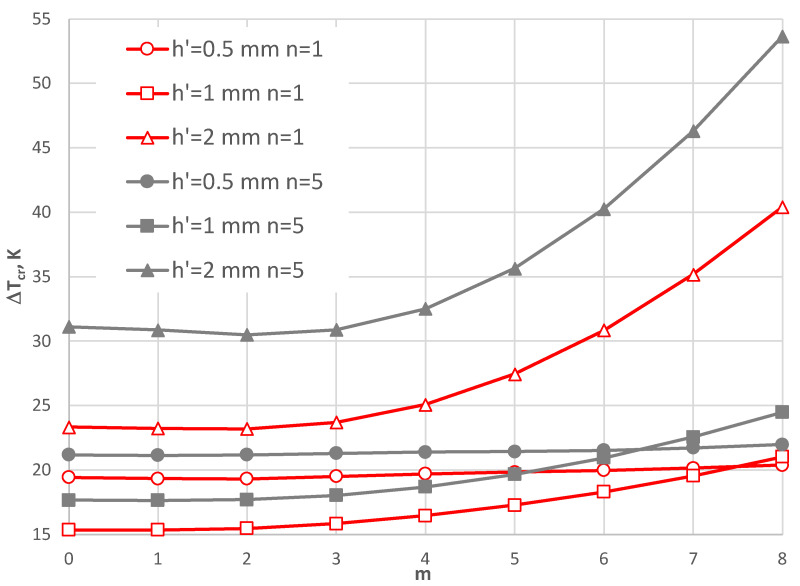
Distribution of the critical temperature difference Δ*T_cr_* versus mode *m* for different facing thicknesses *h’.*

The results of the critical temperature difference Δ*T_cr_* for the plate model supported by the C-C edges are presented in Figure 8, Figure 9, Figure 10, Figure 11, Figure 12 and Figure 13. The plate is subjected to a positive temperature gradient. Figure 8 shows the distribution of Δ*T_cr_* for different numbers *m* of the circumferential waves. The results were calculated for various numbers *n* of the exponent of Equation (6). The value of the dimensionless radius parameter *ρ_i_* is equal to 0.4. The detailed values of the minimal value of Δ*T_cr_* and the corresponding number of mode *m* are presented in Table 4. The FGM facings decrease the value of Δ*T_cr_*. The smallest value is observed for *n* = 1, where the linear distribution of the material parameters along the radial plate direction exists (Figure 3). Next, the axisymmetric form of the loss of plate stability (*m* = 0) is observed. The results for the material model, described by *n* = 5, where the participation of steel is high, are close to those obtained for steel facings. 

A comparison between the plates with different geometry is shown in Figure 8, Figure 9 and Figure 10. For higher and smaller values of the inner radius of the plate hole (*ρ_i_* = 0.5 and *ρ_i_* = 0.3), the minimal value of Δ*T_cr_* is still for *n* = 1. For the smaller value of the inner plate radius *ρ_i_* = 0.3, the results for Δ*T_cr_* are more similar (Figure 10). It is observed for plates with mode *m* = 0 or 1 with an FGM distribution that *n* = 5 and 0.2 and *n* = 2 and 0.5. There are no great changes (see the *E_r_* distribution in Figure 3). With an increase in the plate hole (see Figure 9 for *ρ_i_* = 0.5), the values of the critical temperature difference Δ*T_cr_* are closer for the homogeneous plates made of steel or aluminum and composite plate with *n* equal to 5. 

The influence of the different thicknesses of the facings on the temperature difference Δ*T_cr_* is shown in Figure 8, Figure 11, Figure 12 and Figure 13. The effect of the facings thickness *h*’ provides the temperature differences. With an increase in *h*’ (see Figure 12 for *h*’ = 2 mm), the distribution of the values of Δ*T_cr_* is more regular. The greater values of Δ*T_cr_* are seen for plates with steel facings or those made of composite material with *n* = 5. The smaller values of Δ*T_cr_* are for aluminum facings or composite ones with less participation of steel material for *n* = 0.2. This regularity is not observed for thinner facings of *h*’ = 1 mm (Figure 8) or especially for *h*’ = 0.5 mm (see Figure 11). Whereas, the values of Δ*T_cr_* for plates with aluminum facings are greater than for other plates. The linear distribution of steel and aluminum materials (*n* = 1) influences the minimal values of the critical temperature differences Δ*T_cr_*.

Figure 13 shows a comparison between the composite plates with facings, whose thickness *h*’ is different, and the participation of materials is linear (*n* = 1) or with steel dominating the composite (*n* = 5). The minimal values of Δ*T_cr_* are observed for the thickness of the facings equal to *h*’ = 1 mm. The nonlinear participation of the steel material (*n* = 5), or thicker or thinner facings, increases the values of Δ*T_cr_*.

To summarize, the linear distribution of the two material components, which create the composite facings, is not advantageous for stabilizing the three-layered plate. Creating the structures with higher or less participation of the steel material, defined by exponent *n* = 5 or 0.2, respectively, increases the values of the critical temperature differences Δ*T_cr_*. It should be emphasized that the thickness of the facings is of high importance when undertaking thermal-stability problems. There is a significant difference between plates with thicker and thinner facings.

## 8. SC-SC Plate Model

**Figure 14:**
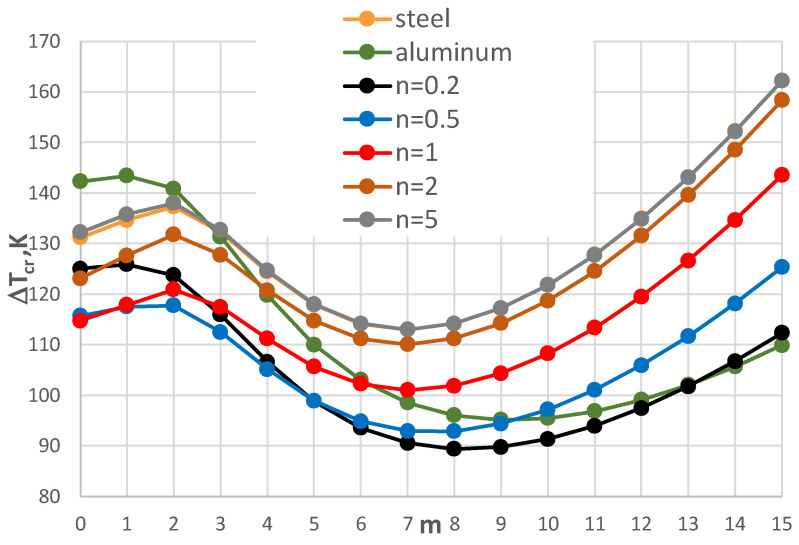
Distribution of the critical temperature difference Δ*T_cr_* versus mode *m* for *h*’ = 1 mm.

**Figure 15:**
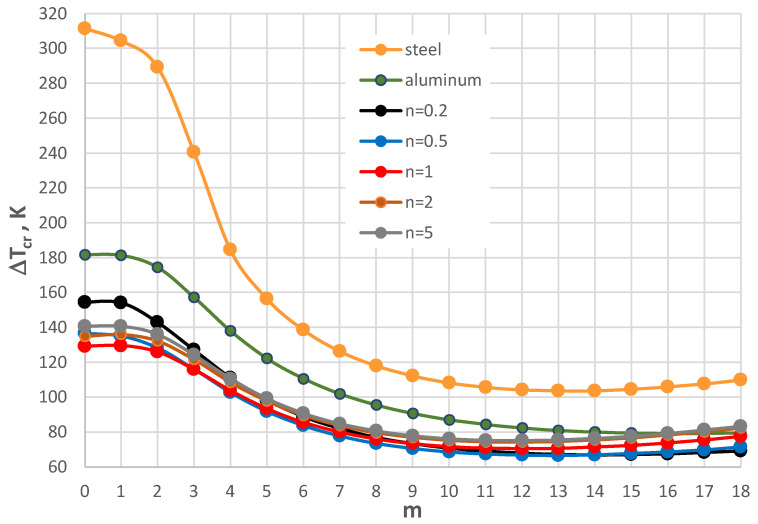
Distribution of the critical temperature difference Δ*T_cr_* versus mode *m* for *h*’ = 0.5 mm.

**Figure 16:**
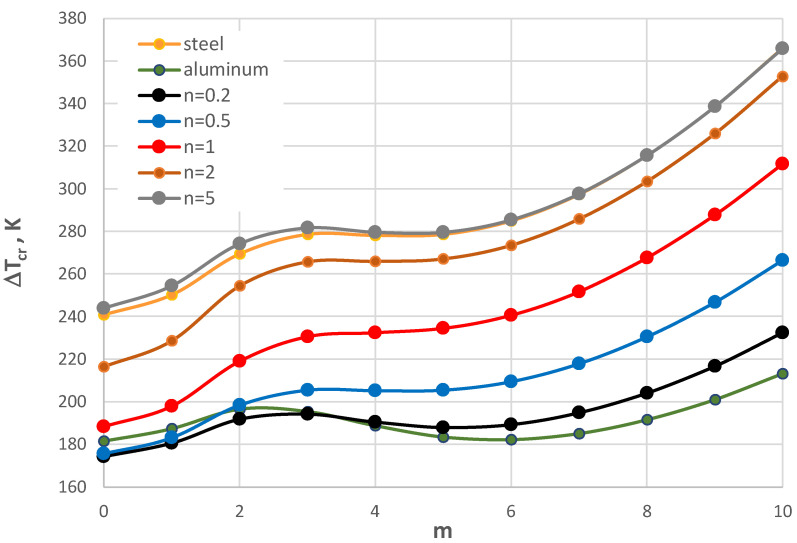
Distribution of the critical temperature difference Δ*T_cr_* versus mode *m* for *h*’ = 2 mm.

**Figure 17:**
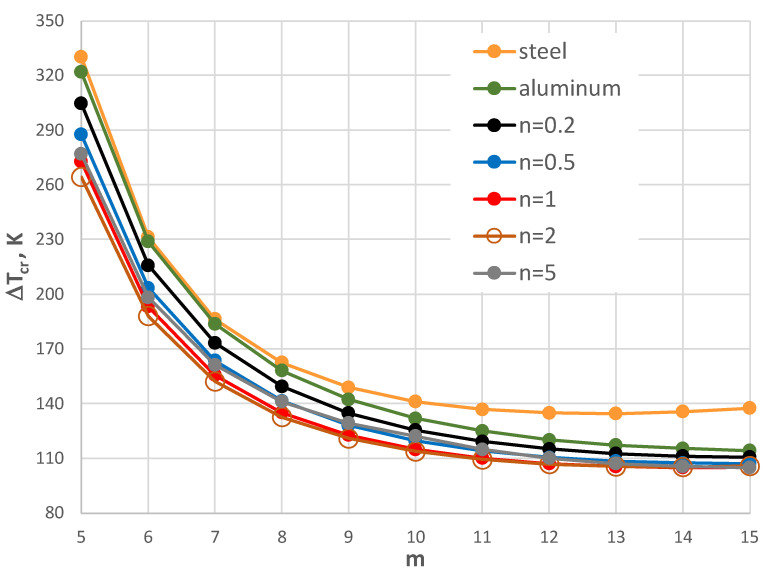
Distribution of the critical temperature difference Δ*T_cr_* versus mode *m* for *h*’ = 1 mm with a plate loaded with a negative temperature gradient.

**Figure 18:**
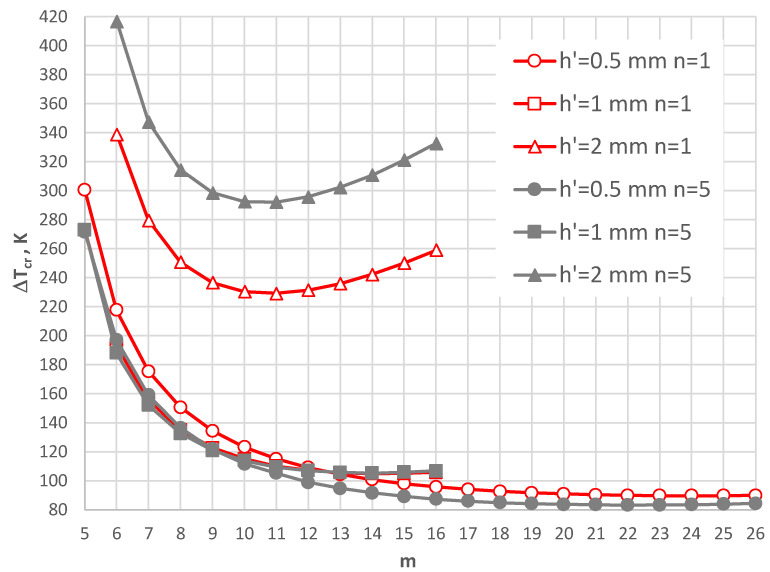
Distribution of the critical temperature difference Δ*T_cr_* versus mode *m* for different facing thickness *h’* with a plate loaded with a negative temperature gradient.

The thermal response of the plate model with the geometry shape expressed by *ρ_i_* = 0.4 and supported by the SC-SC edges is shown in Figure 14, Figure 15, Figure 16, Figure 17 and Figure 18. Figure 14, Figure 15 and Figure 16 present the critical temperature differences Δ*T_cr_*, which depend on buckling mode m for plates with different facing thicknesses, *h*’ = 1, 0.5, and 2 mm, respectively. The plates are subjected to a positive temperature gradient. The exemplary, minimal values of Δ*T_cr_* are presented in Table 5 for the plate with a facing thickness *h*’ equal to 1 mm. The minimal values of Δ*T_cr_* are for a buckling mode with several *m* = 7, 8, and 9 circumferential waves. The minimal values are for the FGM facings characterized by exponent number *n* equal to 0.2. For *n* = 0.2 and 0.5, the values of Δ*T_cr_* are less than for aluminum facings. The facings thickness *h*’ has a large effect on the plate’s thermal response. 

For small thicknesses of the facings, the critical form of the plate buckling is circumferentially waved, but for thicker facings, it is an axisymmetrical (m = 0) one. For plates with very thin facings (i.e., *h*’ = 0.5 mm), all the examined FGM plates lose static stability in a waved form, where the minimal value of Δ*T_cr_* is less than for the homogeneous facings composed of either steel or aluminum. The results for the plates subjected to a negative temperature gradient are shown in Figure 17 and Figure 18. Table 6 presents the values for the homogeneous and the FGM plates with *h*’ = 1 mm. All the minimal values of Δ*T_cr_* for the FGM plates are less than those calculated for the homogeneous plates with steel or aluminum facings. The values are comparable with the level equal to Δ*T_cr_* = 105 K. 

The minimal values of Δ*T_cr_* are for the FGM plates with number *n* equal to 1 and 5. The form of the critical deformation is characterized by a dozen or so waves, *m* = 14, 15, and 16 (see also Figure 17). With an increase in the facing thickness *h*’, the value of Δ*T_cr_* increases (Figure 18). The greater differences between the values Δ*T_cr_* for plates differing with material distribution *n* = 1 and 5 are observed. The buckling shape moves *m* to a form with less circumferential waves. For plates with very thin FGM facings, a high buckling deformation with more than twenty circumferential waves is observed. Here, the values of Δ*T_cr_* are minimal. 

To summarize, it is observable that the facings thickness h’ of the plate structure geometry has an effect on the thermal plate responses and influences the minimal values of Δ*T_cr_*. Moreover, the form of the critical buckling is different for plates with thin or thicker FGM facings. The radial material distribution expressed by the exponent *n* changes the thermal-plate reaction differently, depending upon the thickness of the outer plate layers. Here, the direction of the temperature gradient also has meaning. 

## 9. Conclusions

This paper presented a problem that focused on the evaluation of the static and thermal responses of the composite annular plates with functionally graded material in the facings. The plate is subjected to a temperature field directed in a radial direction from the outer perimeter or to the hole of the plate. The examined annular plate with the FGM facings and homogeneous core, used particularly in mechanical design, creates a new generation of composite structures whose radially, smoothly changing material parameters can improve thermal and mechanical capacity. The analyzed thermal, critical state is defined by the values of the critical temperature differences Δ*T_cr_*, and corresponding with them is the mode buckling m form. The influence of the geometrical and material plate features on the final buckling results is taken into account. Moreover, the support system and the temperature gradient direction are included in the investigations. 

The evaluation was conducted using numerical calculations based on the approximation method and finite differences and then adopted into the author’s program. The presented figures and selected detailed results show the problem in terms of multiple parameters, whose solution depends on the facing thickness, the radial distribution of the FGM, the direction of the temperature gradient, the kind of plate support, and the plate dimensions. For the representative plate with facing thickness *h*’ = 1 mm and dimensionless inner radius equal to *ρ_i_* = 0.4, the minimal values of the critical temperature differences Δ*T_cr_* is, for the clamped–clamped (C-C) plate with linearly variable material parameters, defined by exponent *n* = 1. For the plate whose edges are SC-SC, the slidably clamped plate loses stability for much higher values of temperature differences Δ*T_cr_*. The minimal values for the discussed representative plate are, for example, with the FGM-facing materials, for which aluminum parameters are predominant. Then, *n* is equal to 0.2. The buckling shape is described in terms of several circumferential waves. The gradient of temperature means the minimal values of critical temperature differences, especially for plates with thicker facings. 

The main general conclusions, which are formulated after the presented investigations, are as follows:

- Material and geometrical parameters of the annular sandwich plates specify the values of the critical thermal state.
- The return of the radial temperature gradient changes the responses of the annular plate.
- The linear distribution of the material parameters along the plate radius defined by exponent *n* = 1 for the C-C annular plates is the one for whom the critical temperature difference Δ*T_cr_* is minimal or is close to the smallest. The greater thickness of the facings can cause some disorder.

The analyzed investigation model for the temperature field is fixed and static. In real-world conditions, the plate element can be subjected to variables, which involve a time-dependent temperature field. The dynamic response of the FGM composite plate seems to have interesting scientific and practical applications. It creates a cognition issue that is worth investigating, which will be presented in a future article.

## Figures and Tables

**Table 1:** Geometrical and material plate parameters.

Geometrical Parameters	Data
inner radius *r_i_*	0.15 m, 0.2 m, 0.25 m
outer radius *r_o_*	0.5 m
facing thickness *h*’	0.5 mm, 1 mm, 2 mm
core thickness *h*_2_	5 mm
**Material parameters**	**Data**
Young’s modulus of steel *E_ST_*	210 GPa
Young’s modulus of aluminum *E_AL_*	70 GPa
Poisson’s ratio of steel *ν_ST_*	0.3
Poisson’s ratio of aluminum *ν_AL_*	0.34
linear thermal expansion coefficient of steel *α_ST_*	12 × 10^−6^ 1/K
linear thermal expansion coefficient of aluminum *α_AL_*	24 × 10^−6^ 1/K
FGM facing material exponent *n* (6)	0.2, 0.5, 1, 2, 5
Kirchhoff’s modulus of polyurethane foam as core material *G*_2_	5 MPa

**Table 2:** Critical temperature difference Δ*T_cr_* for the C-C and SC-SC plate models depending on discrete number *N*.

Critical Temperature Differences Δ*T_cr_*, K/Mode *m*						
Plate Model	*n* = 0.5			*n* = 5		
	*N* = 14	*N* = 26	*N* = 35	*N* = 14	*N* = 26	*N* = 35
C-C (Figure 4 and Figure 5)	20.16/8	20.27/8	20.29/8	20.84/6	20.95/6	20.97/6
SC-SC (Figure 6 and Figure 7)	272.82/5	287.65/5	291.21/5	155.40/7	161.17/7	162.49/7

**Table 3:** Critical temperature differences Δ*T_cr_* for two cases, the FDM and FEM plate models, supported by the C-C and SC-SC edges and composed of homogeneous facings made of steel or aluminum.

Critical Temperature Differences Δ*T_cr_*, K/Mode *m*				
Plate Model	Steel		Aluminum	
	FDM	FEM [19]	FDM	FEM [19]
C-C	17.83/0	17.79/0	19.33/0	19.29/0
	17.79/1	17.81/1	19.21/1	19.26/1
SC-SC	131.21/0	131.29/0	142.26/0	142.15/0
	134.62/1	135.08/1	143.48/1	143.92/1
	113.01/7 (117.23/9)	118.11/9	95.12/9 (96.83/11)	101.45/11

**Table 4:** Critical temperature differences Δ*T_cr_* and mode m for the C-C plate model with *h*’ = 1 mm and *ρ_i_* = 0.4 under a positive gradient.

Critical Temperature Differences Δ*T_cr_*, K/Mode *m*						
Steel	Aluminum	*n* = 0.2	*n* = 0.5	*n* = 1	*n* = 2	*n* = 5
17.79/1	19.16/2	17.04/2	15.78/1	15.35/0	15.98/0	17.64/1

**Table 5:** Critical temperature differences Δ*T_cr_* and mode *m* for the SC-SC plate model with h’ = 1 mm.

Critical Temperature Differences Δ*T_cr_*, K/Mode *m*						
Steel	Aluminum	*n* = 0.2	*n* = 0.5	*n* = 1	*n* = 2	*n* = 5
113.01/7	95.12/9	89.38/8	92.93/7	101.08/7	110.11/7	113.05/7

**Table 6:** Critical temperature differences Δ*T_cr_* and mode *m* for the SC-SC plate model for *h*’ = 1 mm with a plate loaded with a negative temperature gradient.

Critical Temperature Differences Δ*T_cr_*, K/Mode *m*						
Steel	Aluminum	*n* = 0.2	*n* = 0.5	*n* = 1	*n* = 2	*n* = 5
134.52/13	114.03/16	110.49/15	107.12/15	104.80/14	105.25/14	104.80/15

## Data Availability

The original contributions presented in the study are included in the article, further inquiries can be directed to the corresponding author/s.

## The List of Abbreviations and Main Parameters

**Abbreviations**
FGM—functionally graded material
FDM—finite difference method
C-C—clamped–clamped plate support system
SC-SC—slidably clamped plate support system
**Main parameters**
*ρ_i_*—dimensionless inner plate radius
*h*’—facing thickness
*h*_2_—core thickness
*E_ST_*—Young’s modulus of steel
*E_AL_*—Young’s modulus of aluminum
*α_ST_*—linear thermal expansion coefficient of steel
*α_AL_*—linear thermal expansion coefficient of aluminum
*n*—FGM facing material exponent, (see Equation (6))
*G*_2_—Kirchhoff’s modulus of polyurethane foam as core material
*T—*temperature
$\Delta T=T_{i}-T_{o}$—temperature difference
Δ*T_cr_*—critical temperature difference
*N*—number of discretization points
*m*—plate buckling mode
